# Urinary angiotensinogen and urinary sodium are associated with blood pressure in normoalbuminuric children with diabetes

**DOI:** 10.1007/s00467-014-2861-0

**Published:** 2014-06-01

**Authors:** Jolanta Soltysiak, Bogda Skowronska, Piotr Fichna, Danuta Ostalska-Nowicka, Witold Stankiewicz, Maria Lewandowska-Stachowiak, Katarzyna Lipkowska, Jacek Zachwieja

**Affiliations:** 1 Department of Pediatric Cardiology and Nephrology, Poznan University of Medical Sciences, 27/33 Szpitalna St., 60-572 Poznan, Poland; 2 Department of Pediatric Diabetes and Obesity, Poznan University of Medical Sciences, Poznan, Poland

**Keywords:** Hypertension, Diabetic kidney disease, Ambulatory blood pressure monitoring

## Abstract

**Background:**

The aim of this study was to evaluate the association between blood pressure (BP) and urinary angiotensinogen excretion (uAGT) and renal sodium excretion (uNa) in children with type 1 diabetes mellitus (DM1).

**Methods:**

The study group consisted of 52 children with DM1 (28 males and 24 females) with albumin/creatinine ratio (ACR) below 30 mg/g and glomerular filtration rate (eGFR) above 90 ml/min/1.73 m^2^. BP was assessed by 24-h ambulatory blood pressure monitoring (ABPM).

**Results:**

The patients showed significantly increased uAGT values with respect to controls (median 0.00 and range 1.76 vs. 0.00 and 0.00 ng/mg, respectively). The significant increase of uAGT was observed even in prehypertensive patients. uAGT concentrations showed positive correlation with systolic and diastolic 24-h BP and with mean arterial pressure (MAP) (*r* = 0.594). uNa values were negatively correlated with BP parameters, uAGT, ACR and eGFR.

**Conclusions:**

An increase in uAGT precedes hypertension (HTN) in normoalbuminuric children with DM1 and may be considered as a new marker of HTN. Decreased sodium excretion seems to be involved in the development of HTN and early renal injury. Both uAGT and uNa are associated with BP in normoalbuminuric diabetic children.

## Introduction

Diabetic kidney disease (DKD) is a leading cause of chronic kidney disease, with a high risk of end-stage renal disease [1, 2]. DKD, previously known as diabetic nephropathy, is defined as persistent proteinuria greater than 500 mg/24 h or albuminuria greater than 300 mg/24 h, and is usually associated with hypertension (HTN) and decreased glomerular filtration rate [3]. The first clinical sign of incipient nephropathy secondary to diabetes is microalbuminuria [4, 5]. However, some patients are able to develop DKD without preceding microalbuminuria or can reduce renal function even during the microalbuminuria stage [6, 7]. Therefore, a more sensitive and specific marker for incipient DKD is needed. In clinical and experimental trials, it has been shown that activation of the intrarenal renin-angiotensin system (RAS) has a potential role in the mechanism and progression of DKD [8–10]. The excretion of urinary angiotensinogen could be a potential biomarker of intrarenal RAS status in clinical and experimental type 1 diabetes [11–13]. Angiotensinogen (AGT) is the only known substrate for renin, which is the rate-limiting enzyme of the RAS [14]. AGT is synthesized in the liver and proximal tubule. The origin of urinary AGT (uAGT) is not fully known. Some authors conclude that uAGT derives only from the kidneys and reflects intrarenal activation of the RAS [15–17]. However, Matsusaka et al. concluded that in non-diabetic mice, the primary source of renal AGT protein and angiotensin II is liver AGT, which is filtered by the glomeruli and reabsorbed by the proximal tubule through megalin-intact cells [18]. Disruption of the filtration barrier in a transgenic mouse model increased both tubular and urinary AGT without any increase in renal renin activity.

On the other hand, it has been shown that in diabetic rats, overexpression of AGT in the proximal tubule may result from hyperglycemia and may lead to tubular apoptosis, tubulointerstitial fibrosis and HTN [19, 20]. HTN is a risk factor and may accelerate the progression of microvascular and macrovascular complications including DKD [21]. Identification of individuals at risk of developing HTN and renal disease is of cardinal importance to retard the progression of renal injury in diabetes [21]. AGT is important not only in the control of arterial pressure, but also in the control of renal sodium excretion [16]. Enhanced tubular sodium reabsorption is stimulated by intrarenal angiotensin II as indicated by proximal tubular AGT, and may contribute to the genesis of HTN [16, 22–24].

In the present study, we investigated uAGT and urinary sodium excretion (uNa) in relation to BP in children with type 1 diabetes mellitus (DM1) not presenting diabetic kidney disease; that is, with normoalbuminuria and glomerular filtration rate above 90 ml/min/1.73 m^2^.

## Material and methods

### Patient and control groups

The study group consisted of 52 children with DM1 (28 males and 24 females) with a mean age of 14.39 ± 2.49 years. The average time of treatment was 7.86 ± 14.76 years. Children with infections, inflammatory states, proteinuria, glycosuria or any abnormal urine analysis or renal impairment were excluded to avoid potential confounding factors. None of the patients was overweight or obese. We used relative body mass index (RBMI) to estimate body fat content. RBMI was defined as actual BMI divided by ideal (50th percentile) BMI for specific age and sex. To avoid the impact of very low birth weight (VLBW) on uAGT concentration, children with birth weight below 1,500 g were excluded from the study [25]. All patients had an estimated glomerular filtration rate (eGFR) above 90 ml/min/1.73 m^2^, according to the Filler formula [26] and normal urinary albumin excretion (< 30 mg/g) defined by the albumin/creatinine ratio (ACR). Glomerular hyperfiltration was recognized above 135 ml/min/1.73 m^2^, calculated as the sum of mean value plus two standard deviations (SD) [27]. Screening for microalbuminuria (MA) was performed three times in first early morning urine sample for 3 months. The urine sample collected on the same morning as the blood sample was chosen for statistical analysis. Long-term glycemic control was based on hemoglobin A1c (HbA1c) levels [28]. To better assess glycemic control, patients were categorised by HbA1c values into; a group with ideal glycemic control (< 6.5 % HbA1c), optimal (< 7.5 % HbA1c), suboptimal (7.5–9.0 % HbA1c) and poor (> 9.0 % HbA1c) [28]. None of the patients received RAS blockades. All subjects continued on free sodium intake and no medication other than insulin was allowed.

The control group consisted of 20 healthy, age-matched and gender-matched adolescents hospitalized due to suspected urinary tract defects (congenital abnormalities). Patients with congenital abnormalities, as well as infections, kidney diseases, diabetes mellitus and HTN, were excluded based on clinical examination, laboratory tests and abdominal ultrasonography. Urine and blood samples were collected on the same morning and frozen immediately.

The study protocol was approved by the local Ethics Committee, and every participant and his/her parents/legal guardians gave fully informed consent to take part in the study. The study was conducted according to the principles expressed in the Second Declaration of Helsinki.

### 24-hour ambulatory blood pressure monitoring (ABPM)

ABPM was performed using an oscillometric device (SpaceLabs 90217) approved by the European Society of Hypertension [29]. The monitor was programmed to measure BP every 20 min during the day (7 am–10 pm), and every 30 min during the night (10 pm–7 am). The parents and children were instructed to keep a diary of daily activities during the ABPM measurement. However, in order to compare with the normative values for ABPM, we defined the daytime period as 8 am to 8 pm, and the nighttime period as 12 pm to 6 am [30, 31]. The cuff size was determined by measurement of the mid-arm circumference, and was approximately 40 % of the arm circumference [26]. The cuff was placed on the non-dominant arm. The patients were instructed to avoid vigorous physical exercise during ABPM measurement, but to follow their usual daily activities. A minimum of 40 recordings was required to consider the ABPM as valid [30]. The following ABPM parameters were analyzed: mean arterial pressure (MAP), systolic/diastolic BP over 24 h, during daytime and nighttime. The diurnal BP rhythmicity was assessed by the ratio between the day and night (D/N) BP for MAP. A D/N ratio of ≥ 1.1 was considered as normal dipping status, whereas non-dipping status was defined as a D/N ratio *<*1.1.

Patients were considered as hypertensive if their MAP, systolic and diastolic BP were above the 95th percentile, regardless of BP load and dipping/non-dipping status. Prehypertensive children (preHTN) showed BP below the 95th percentile, while normal BP (nBP) was recognized as below the 90th percentile.

### Measurements

AGT was measured in urine using a commercially available enzyme-linked immunosorbent assay (ELISA) kit in accordance with the manufacturer’s instructions (Uscn Life Science Inc., Houston, USA). Inter-assay and intra-assay coefficient variations were 5–10 %. Urine samples were collected in the morning and immediately stored at −70 °C. All urine specimens were used within 3 months after collection. To avoid differences in spot urine, uAGT was corrected for urinary creatinine. uNa and common biochemical parameters were determined in the clinical laboratory of the Poznan Medical University Hospital. Urinary albumin and creatinine concentrations were measured using an automated analyzer (Bayer).

### Statistical analysis

Results of the studied biomarkers were non-normally distributed and nonparametric testing was used to compare all concentrations between the groups (Mann–Whitney test). Values that followed a non-normal distribution are expressed as mean ± standard deviation (SD). The relationship between two variables was assessed by Spearman’s rank correlation coefficient. The level of statistical significance was < 0.05.

## Results

**Fig. 1:**
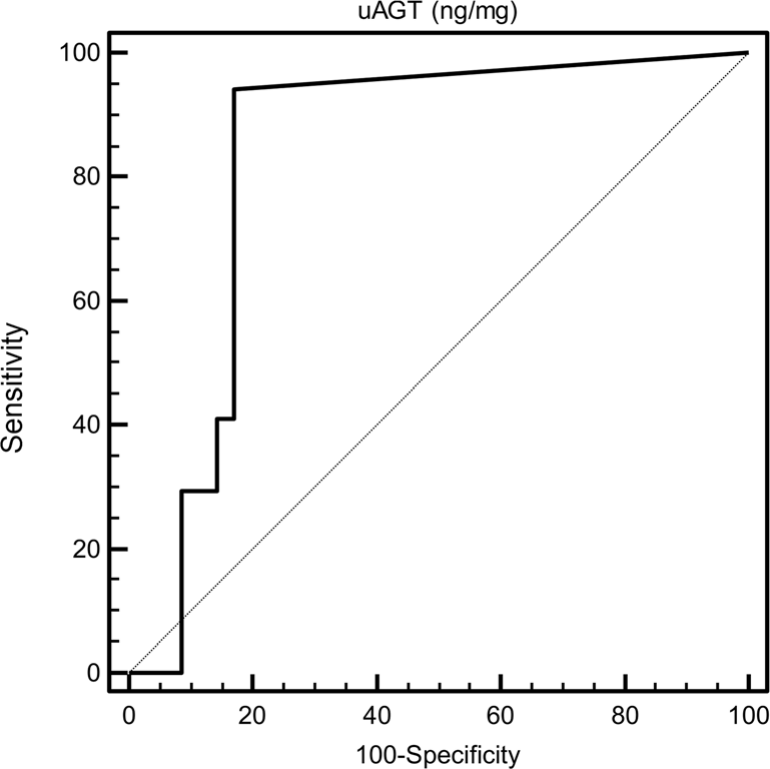
Receiver operating characteristic (ROC) curves of uAGT considering the presence of hypertension (≥ 95 pc) in normoalbuminuric children with diabetes as status variable. The AUC for uAGT was 0.833 (CI: 0.704–0.922). The best cutoff value for the identification of hypertension in diabetes was found to be above 0 ng/mg, with a sensibility of 94.12 (CI: 71.3–99.9) and a specificity of 82.86 (CI: 68.4–93.4), *uAGT* urinary angiotensin

The demographic and clinical data of the study group and controls are given in Table 1. Children with DM1 showed increased uAGT values with respect to controls (median 0.00 and range 1.76 vs 0.00 and 0.00 ng/mg). ACR, eGFR and uNa levels did not differ between the study groups. The comparison of children with normal BP, preHTN and HTN revealed a significant increase of uAGT in preHTN patients, as well as in HTN, when compared to controls (Table 2, Fig. 1). Changes of ACR, eGFR and uNa levels were not significant (Table 2).

**Table 1:** The demographic and clinical data of the study group and controls

Parameters	Diabetes, *n* = 52		Controls, *n* = 20		p
	Median	Range	Median	Range	
uAGT (ng/mg)	0.00	1.76	0.00	0.00	0.015
Age (yr)	14.56	8.73	12.08	10.61	NS
RBMI (%)	104.7	15.96	101.81	16.66	NS
eGFR (ml/min/1.73 m2)	111.44	140.06	101.27	83.99	NS
HbA1c (%)	7.95	9.00	5.45	0.80	<0.001
ACR (mg/g)	8.09	24.46	6.25	7.61	NS
uNa (mmol/l)	100.95	235.10	102.80	141.70	NS
sNa (mmol/l)	140.50	14.00	139.50	6.00	NS
FE_Na_	0.33	1.06	0.25	0.48	NS

*p* confidence level, *NS* non-significant, *na* not applicable, *SD* standard deviation, *uAGT* urinary AGT corrected for urinary creatinine (ng/mg), *uNa* urinary sodium, *sNa* serum sodium, *RBMI* relative body mass index, *eGFR* estimated glomerular filtration rate, *ACR* albumin/creatinine ratio

**Table 2:** The comparison of diabetic children to controls by blood pressure groups

Parameters	nBP; *n* = 28		preHA; *n* = 7		HA; *n* = 17		Controls		p*	p**	p***
	Median	Range	Median	Range	Median	Range	Median	Range			
uAGT (ng/mg)	0.00	1.76	0.04	0.88	0.02	0.35	0.00	0.00	NS	0.009	< 0.001
eGFR (ml/min/1.73 m^2^)	105.77	83.52	105.77	45.54	122.04	134.22	101.27	83.99	NS	NS	NS
ACR (mg/g)	6.12	23.79	7.92	20.38	12.81	24.28	6.25	7.61	NS	NS	NS
uNa (mmol/l)	112.00	214.00	91.50	162.10	83.15	143.50	102.80	141.70	NS	NS	NS
sNa (mmol/l)	141.00	14.00	138.00	8.00	140.00	10.00	139.50	6.00	NS	NS	NS
FE_Na_	0.40	0.87	0.51	0.63	0.22	0.58	0.25	0.48	NS	NS	NS

p*—confidence level between nBP and Controls; p**—confidence level between preHA and Controls

p***—confidence level between HA and Controls

*nBP* normal blood pressure, *preHA* prehypertensive, *HA* hypertensive, *uNa* urinary sodium, *sNa* serum sodium; *SD* standard deviation, *uAGT* urinary AGT corrected for urinary creatinine (ng/mg), *eGFR* estimated glomerular filtration rate, *ACR* albumin/creatinine ratio

uAGT levels showed a positive correlation with BP values, both systolic and diastolic 24-h BP, with the strongest relationship with MAP (*r* = 0.594). The D/N ratio demonstrated negative correlation with uAGT values (*r* = −0.279). No correlations between uAGT and ACR, eGFR or HbA1c, as well as diabetes duration were found. There was no relationship between uAGT and HbA1c in different subgroups, including those with different diabetic control. In contrast, ACR, eGFR, and HbA1c, as well as uAGT, were negatively correlated with uNa (Table 3). Moreover, uNa demonstrated a negative relationship with BP parameters, especially systolic BP and D/N ratio.

**Table 3:** The correlations of uAGT and uNa values with blood pressure and clinical parameters in the diabetic group

Parameters	uAGT (ng/mg)	uNa (mmol/l)
24S	*r* = 0.538	*r* = −0.346
	*p* < 0.001	*p* = 0.012
24R	*r* = 0.485	NS
	*p* < 0.001	
24MAP	*r* = 0.594	*r* = −0.293
	*p* < 0.001	*p* = 0.035
D/N ratio	*r* = −0.279	*r* = 0.419
	*p* = 0.042	*p* = 0.002
Diabetes duration (yr)	NS	*r* = −0.401
		*p* = 0.003
eGFR (ml/min/1.73 m2)	NS	*r* = −0.338
		*p* = 0.011
HbA1c (%)	NS	*r* = −0.334
		*p* = 0.009
ACR (mg/g)	NS	*r* = −0.329
		*p* = 0.008
uNa (mmol/l)	*r* = −0.379	na
	*p* = 0.009	
sNa (mmol/l)	NS	NS
FE_Na_	*r* = −0.337	na
	*p* = 0.006	

*p* confidence level, *NS* non-significant, *na* not applicable, *D/N ratio* the ratio between day and night (D/N) BP for MAP, *uAGT* urinary AGT corrected for urinary creatinine (ng/mg), *uNa* urinary sodium, *sNa* serum sodium, *ACR* albumin/creatinine ratio, *AGT* angiotensin

There was no significant correlation between BP parameters, that is, between systolic BP, diastolic BP, D/N ratio and eGFR, ACR.

Thirteen children (25 %) presented glomerular hyperfiltration.

### Receiver operating characteristic (ROC) analysis of uAGT

ROC analysis was performed in order to define the diagnostic profile of uAGT in identifying hypertensive patients (above 95th percentile) among all diabetic children with normal albumin excretion. To this end, uAGT showed a good diagnostic profile, describing an AUC of 0.833 (CI: 0.704–0.922) with a best cutoff value of > 0 ng/mg (sensitivity 94.12 %; specificity 82.86 %; see Table 4; Fig. 1).

**Table 4:** ROC analysis of urine angiotensin (uAGT) values

uAGT (ng/mg)	Sensitivity (95 % CI)	Specificity (95 % CI)	+LR	-LR
≥ 0	100.00 (80.5–100.0)	0.00 (0.0–10.0)	1.00	–
> 0^a^	94.12 (71.3–99.9)	82.86 (68.4–93.4)	5.49	0.071
> 0.03	41.18 (18.4–67.1)	82.86 (66.4–93.4)	2.40	0.71
> 0.353	0.00 (0.0–19.5)	91.43 (76.9–98.2)	0.00	1.09
> 1.7591	0.00 (0.0–19.5)	100.00 (90.0–100.0)	–	1.00

Best cutoff value for the identification of hypertension in normoalbuminuric children with diabetes: > 0 mg/mg. Area under the ROC curve = 0.833; standard error = 0.0602; 95 % confidence interval = 0.704–0.922. +LR = positive likelihood ratio

*−LR* negative likelihood ratio

^a^Best uAGT cutoff value

## Discussion

The present study shows that in normoalbuminuric children with DM1 and with glomerular filtration above 90 ml/min/1.73 m^2^, there is a significant increase in the level of uAGT. This suggests that uAGT may serve as an early marker of renal involvement in patients with diabetes, and indicates that early activation of RAS may precede all other abnormalities typically required to diagnose DKD. Furthermore, uAGT correlated positively with BP. Children with normal BP—that is, below the 90th percentile—did not show elevated uAGT. The significant increase in uAGT was observed even in prehypertensive patients. These results indicate the potential association of uAGT with HTN in diabetic children, with an additional predictive value to detect prehypertensive children. Moreover, the ROC analysis revealed a good diagnostic profile of uAGT in identifying hypertensive patients, and showed that uAGT may be considered as a new marker of HTN in DM1. The association of 24-h systolic and diastolic BP with uAGT in diabetic children has not been previously studied. However, a positive relationship between uAGT and BP has been demonstrated in many reports in hypertensive, but non-diabetic patients, including adolescents [16, 20, 23, 32–34]. It has been shown that increased uAGT, through increased angiotensin II levels, leads to salt-sensitive HTN via salt retention [16, 20, 22, 23, 32, 34]. In this study, the inverse correlation between uAGT and uNa was demonstrated. This might suggest that enhanced sodium reabsorption is caused by elevated angiotensin II, reflected by the increased level of uAGT. However, in hypertensive, non-diabetic patients, it was shown that uAGT excretion was higher, with greater uNa resulting from higher sodium intake [22, 23, 32]. The authors concluded that increased dietary sodium stimulates the expression of AGT in the proximal tubule and is associated with clinical and ambulatory HTN [20, 32, 35, 36].

In diabetes, elevated uAGT and enhanced intrarenal RAS activity may be caused by hyperglycemia without being affected by sodium intake [9, 10, 19]. This is a potential mechanism for the development of HTN in diabetic patients. However, in the early stages of diabetes type 1, the influence of enhanced sodium reabsorption stimulated only by hyperglycemia seems to play an important role. The increase in glucose and sodium reabsorption through enhanced expression of the Na/glucose co-transporter (SGLT) in the proximal tubule decreases uNa. Reduced uNa concentration in distal tubular fluid at the macula densa enhances glomerular filtration through the tubuloglomerular feedback (TGF) system [37, 38]. This is a typical early signal of renal involvement in diabetes. In the present study, 25 % of diabetic children presented glomerular hyperfiltration, and this may result from the increase in glucose and sodium reabsorption through enhanced expression on SGLT in proximal tubules.

Taken together, arterial HTN in DM1 might be caused by increased sodium retention resulting from two independent mechanisms: increased expression of the Na/glucose co-transporter, and increased angiotensin II levels. Might this, therefore, influence the antihypertensive policy in patients with DM1 using intrarenal RAS blockers or SGLT blockers? The question needs further investigation. However, a recent large-scale clinical study has reported that RAS inhibitors delay renal impairment caused by diabetic nephropathy, and in the present study, RAS blockers were administered to hypertensive patients [39].

It should be emphasized that in this study, uNa levels within the whole study group did not differ from the controls. Similar observations have been reported by other authors, however for adult populations with DM1 [40]. In our children with early diabetic complications, decreased uNa concentration was observed. Children with HTN, as well as those with higher ACR, eGFR and HbA1c values, and longer duration of the disease showed lower levels of uNa. In the literature there is little information concerning the relationship between sodium excretion, albuminuria and renal damage, and these observations require further evaluation.

Interestingly, there was no significant correlation between uAGT and ACR as well as eGFR. Positive correlations between uAGT, ACR and proteinuria have been shown in other studies, but in adult hypertensive patients without diabetes [23]. In type 1 diabetes, these relations were not demonstrated and it was concluded that elevated uAGT in prealbuminuric phase patients is not simply a nonspecific consequence of proteinuria [11, 12]. In non-diabetic mice, it has been shown that filtered AGT was reabsorbed by the proximal tubule [18]; thus, increased levels of uAGT in diabetic children might also result from lack of AGT reabsorption by injured proximal tubules. This may suggest that increased uAGT is an early sign of kidney damage in DM1, before the onset of microalbuminuria.

This study has some limitations. Firstly, it is a relatively small, but homogeneous group. Secondly, the impact of sodium intake was not studied; however, all subjects continued on free sodium intake. Next, intrarenal and serum RAS activity was not measured, but in many previous reports it was clearly stated that urinary AGT reflects intrarenal RAS activity only. Moreover, the exchangeable sodium should be assessed to show sodium retention; however, this exceeded the scope of our study.

In conclusion, the results of our study suggest that in children with DM1, increased levels of uAGT may reflect early renal involvement, before the onset of microalbuminuria. An increase in uAGT precedes HTN, and may be considered as a new marker of HTN in diabetic children with normal urinary albumin excretion. Decreased sodium excretion seems to be involved in the development of HTN and early renal injury. Both uAGT and uNa are associated with BP in normoalbuminuric diabetic children.

## References

[1] Jha V, Garcia-Garcia G, Iseki K, Li Z, Naicker S, Plattner B, Saran R, Wang AY, Yang CW (2013). Chronic kidney disease: global dimension and perspectives. Lancet.

[2] The Diabetes Control and Complications Trial Research Group (1993). The effect of intensive treatment of diabetes on the development and progression of long-term complications in insulin-dependent diabetes mellitus. N Engl J Med.

[3] Donaghue KC, Chiarelli F, Trotta D, Allgrove J, Dahl-Jorgensen K (2009). Microvascular and macrovascular complications associated with diabetes in children and adolescents. Pediatr Diabetes.

[4] Perkins BA, Krolewski AS (2005). Early nephropathy in type 1 diabetes: a new perspective on who will and who will not progress. Curr Diab Rep.

[5] Eknoyan G, Hostetter T, Bakris GL, Hebert L, Levey AS, Parving HH, Steffes MW, Toto R (2003). Proteinuria and other markers of chronic kidney disease: a position statement of the national kidney foundation (NKF) and the national institute of diabetes and digestive and kidney diseases (NIDDK). Am J Kidney Dis.

[6] Dalla Vestra M, Saller A, Bortoloso E, Mauer M, Fioretto P (2000). Structural involvement in type 1 and type 2 diabetic nephropathy. Diabetes Metab.

[7] Perkins BA, Ficociello LH, Silva KH, Finkelstein DM, Warram JH, Krolewski AS (2003). Regression of microalbuminuria in type 1 diabetes. N Engl J Med.

[8] Taguma Y, Kitamoto Y, Futaki G, Ueda H, Monma H, Ishizaki M, Takahashi H, Sekino H, Sasaki Y (1985). Effect of captopril on heavy proteinuria in azotemic diabetics. N Engl J Med.

[9] Nagai Y, Yao L, Kobori H, Miyata K, Ozawa Y, Miyatake A, Yukimura T, Shokoji T, Kimura S, Kiyomoto H, Kohno M, Abe Y, Nishiyama A (2005). Temporary angiotensin II blockade at the prediabetic stage attenuates the development of renal injury in type 2 diabetic rats. J Am Soc Nephrol.

[10] Yoo TH, Li JJ, Kim JJ, Jung DS, Kwak SJ, Ryu DR, Choi HY, Kim JS, Kim HJ, Han SH, Lee JE, Han DS, Kang SW (2007). Activation of the renin-angiotensin system within podocytes in diabetes. Kidney Int.

[11] Kamiyama M, Zsombok A, Kobori H (2012). Urinary angiotensinogen as a novel early biomarker of intrarenal renin-angiotensin system activation in experimental type 1 diabetes. J Pharmacol Sci.

[12] Saito T, Urushihara M, Kotani Y, Kagami S, Kobori H (2009). Increased urinary angiotensinogen is precedent to increased urinary albumin in patients with type 1 diabetes. Am J Med Sci.

[13] Ogawa S, Kobori H, Ohashi N, Urushihara M, Nishiyama A, Mori T, Ishizuka T, Nako K, Ito S (2009). Angiotensin II Type 1 receptor blockers reduce urinary angiotensinogen excretion and the levels of urinary markers of oxidative stress and inflammation in patients with type 2 diabetic nephropathy. Biomark Insights.

[14] Castrop H, Höcherl K, Kurtz A, Schweda F, Todorov V, Wagner C (2010). Physiology of kidney renin. Physiol Rev.

[15] Kobori H, Nishiyama A, Harrison-Bernard LM, Navar LG (2003). Urinary angiotensinogen as an indicator of intrarenal Angiotensin status in hypertension. Hypertension.

[16] Ying J, Stuart D, Hillas E, Gociman BR, Ramkumar N, Lalouel JM, Kohan DE (2012). Overexpression of mouse angiotensinogen in renal proximal tubule causes salt-sensitive hypertension in mice. Am J Hypertens.

[17] Ramkumar N, Stuart D, Ying J, Kohan DE (2013). A possible interaction between systemic and renal angiotensinogen in the control of blood pressure. Am J Hypertens.

[18] Matsusaka T, Niimura F, Shimizu A, Pastan I, Saito A, Kobori H, Nishiyama A, Ichikawa I (2012). Liver angiotensinogen is the primary source of renal angiotensin II. J Am Soc Nephrol.

[19] Vidotti DB, Casarini DE, Cristovam PC, Leite CA, Schor N, Boim MA (2004). High glucose concentration stimulates intracellular renin activity and angiotensin II generation in rat mesangial cells. Am J Physiol Renal Physiol.

[20] Ramkumar N, Kohan DE (2013). Proximal tubule angiotensinogen modulation of arterial pressure. Curr Opin Nephrol Hypertens.

[21] Raile K, Galler A, Hofer S, Herbst A, Dunstheimer D, Busch P, Holl RW (2007). Diabetic nephropathy in 27,805 children, adolescents, and adults with type 1 diabetes: effect of diabetes duration, A1C, hypertension, dyslipidemia, diabetes onset, and sex. Diabetes Care.

[22] Fukuda M, Urushihara M, Wakamatsu T, Oikawa T, Kobori H (2012). Proximal tubular angiotensinogen in renal biopsy suggests nondipper BP rhythm accompanied by enhanced tubular sodium reabsorption. J Hypertens.

[23] Kobori H, Urushihara M, Xu JH, Berenson GS, Navar LG (2010). Urinary angiotensinogen is correlated with blood pressure in men (Bogalusa Heart Study). J Hypertens.

[24] Chiarelli F, Trotta D, Verrotti A, Mohn A (2002). Treatment of hypertension and microalbuminuria in children and adolescents with type 1 diabetes mellitus. Pediatr Diabetes.

[25] Nishizaki N, Hirano D, Nishizaki Y, Fujinaga S, Nagata S, Ohtomo Y, Kaneko K, Shimizu T (2013). Increased urinary angiotensinogen is an effective marker of chronic renal impairment in very low birth weight children. Clin Exp Nephrol.

[26] National High Blood Pressure Education Program Working Group on High Blood Pressure in Children and Adolescents (2004). The fourth report on the diagnosis, evaluation, and treatment of high blood pressure in children and adolescents. Pediatrics.

[27] Marre M, Hallab M, Roy J, Lejeune JJ, Jallet P, Fressinaud P (1992). Glomerular hyperfiltration in type 1, type 2, and secondary diabetes. J Diabet Complicat.

[28] Rewers M, Pihoker C, Donaghue K, Hanas R, Swift P, Klingensmith GJ (2009). Assessment and monitoring of glycemic control in children and adolescents with diabetes. Pediatr Diabetes.

[29] Amoore JN, Dewar D, Gough K, Padfield PL (2005). Do SpaceLabs ambulatory non-invasive blood pressure recorders measure blood pressure consistently over several years use?. Blood Press Monit.

[30] Soergel M, Kirschstein M, Busch C, Danne T, Gellermann J, Holl R, Krull F, Reichert H, Reusz GS, Rascher W (1997). Oscillometric twenty-four-hour ambulatory blood pressure values in healthy children and adolescents: a multicenter trial including 1141 subjects. J Pediatr.

[31] Wühl E, Witte K, Soergel M, Mehls O, Schaefer F, German Working Group on Pediatric Hypertension (2002). Distribution of 24-h ambulatory blood pressure in children: normalized reference values and role of body dimensions. J Hypertens.

[32] Zou J, Li Y, Li FH, Wei FF, Wang JG (2012). Urinary angiotensinogen excretion and ambulatory blood pressure. J Hypertens.

[33] Kuroczycka-Saniutycz E, Wasilewska A, Sulik A, Milewski R (2013). Urinary angiotensinogen as a marker of intrarenal angiotensin II activity in adolescents with primary hypertension. Pediatr Nephrol.

[34] Franco M, Tapia E, Santamaría J, Zafra I, García-Torres R, Gordon KL, Pons H, Rodríguez-Iturbe B, Johnson RJ, Herrera-Acosta J (2001). Renal cortical vasoconstriction contributes to development of salt-sensitive hypertension after angiotensin II exposure. J Am Soc Nephrol.

[35] Kobori H, Harrisom-Bernard LM, Navar LG (2002). Urinary excretion of angiotensinogen reflects intrarenal angiotensinogen production. Kidney Int.

[36] Lantelme P, Rohrwasser A, Gociman B, Hillas E, Cheng T, Petty G, Thomas J, Xiao S, Ishigami T, Herrmann T, Terreros DA, Ward K, Lalouel JM (2002). Effects of dietary sodium and genetic background on angiotensinogen and Renin in mouse. Hypertension.

[37] Vallon V, Blantz R, Thomson S (2005). The salt paradox and its possible implications in managing hypertensive diabetic patients. Curr Hypertens Rep.

[38] Vallon V, Blantz RC, Thomson S (2003). Glomerular hyperfiltration and the salt paradox in early [corrected] type 1 diabetes mellitus: a tubulo-centric view. J Am Soc Nephrol.

[39] Wühl E, Schaefer F (2011). Managing kidney disease with blood-pressure control. Nat Rev Nephrol.

[40] Feldt-Rasmussen B, Mathiesen ER, Deckert T, Giese J, Christensen NJ, Bent-Hansen L, Nielsen MD (1987). Central role for sodium in the pathogenesis of blood pressure changes independent of angiotensin, aldosterone and catecholamines in type 1 (insulin-dependent) diabetes mellitus. Diabetologia.

